# Effect of ganciclovir for the treatment of severe cytomegalovirus-associated pneumonia in children without a specific immunocompromised state

**DOI:** 10.1186/1471-2334-13-424

**Published:** 2013-09-09

**Authors:** Thanh Thi Mai Doan, Thuy Thi Bich Phung, Hung Viet Pham, Son Hong Pham, Liem Thanh Nguyen

**Affiliations:** 1 Vietnam National Hospital of Pediatrics, Hanoi, Vietnam; 2 Research Institute for Child Health, Hanoi, Vietnam; 3 Respiratory Department, National Hospital of Pediatrics, No. 18, Alley 897, La Thanh Street, Dong Da District, Hanoi, Vietnam

**Keywords:** Cytomegalovirus-associated pneumonia, Ganciclovir, Children

## Abstract

**Background:**

This study aimed to evaluate the effectiveness of gancyclovir (GCV) treatment for severe cytomegalovirus (CMV)-associated pneumonia in immunocompetent children.

**Method:**

We enrolled patients with CMV-associated severe pneumonia admitted to the Vietnam National Hospital of Pediatrics, Hanoi, Vietnam, from January 2010 to December 2011. On admission, though respiratory bacteria and viruses were not detected in tracheal aspirates, more than 5 × 10^3^ copies/mL of CMV-DNA were detected in both tracheal aspirates and in blood plasma. GCV was given intravenously at a dose of 10 mg/kg/24 h for a duration of 14 days at most. The dose was then reduced to 5 mg/kg/24 h until CMV-DNA was not detected in plasma. The main study variables included clinical symptoms, complete blood count, hepatic and renal function, chest X-ray, CMV viral load, duration of GCV treatment and outcome.

**Results:**

Forty-three patients were enrolled in the study. The median age of patients was 57 (interquartile range [IQR] 45–85) days. Clinical and laboratory findings included anemia (67.4%), leukocytosis (90.7%), hepatosplenomegaly (60.5%), elevated liver enzymes (74.4%), decreased ratio of CD4: CD8-positive T lymphocytes (69.4%), and decreased serum IgG concentration (25.7%). The median duration of GCV treatment was 12 days (IQR 7-21). Thirty-seven patients (86.0%) showed normal chest X-rays at the end of treatment. One infant died (2.3%); the other children (97.7%) were discharged in good condition. There was no severe toxicity associated with GCV treatment.

**Conclusion:**

GCV is safe and effective for the treatment of severe CMV-associated pneumonia in children.

## Background

Cytomegalovirus (CMV) is a common cause of acute lower respiratory infection, particularly in immunocompromised hosts, and is associated with high mortality [1-3]. While CMV infection in immunocompetent individuals has traditionally been considered a benign and self-limited disease, a few studies have reported the clinical manifestations and treatment of CMV infection among immunocompetent patients [4-8]. However, little is known about the clinical and laboratory features and the role of antiviral medications in the treatment of severe CMV-associated pneumonia in children who are not immunocompromised.

The aim of the present study was to evaluate the effectiveness of GCV in severe CMV-associated pneumonia treatment in children.

## Methods

### Patients and case definitions

This study was carried out from January 2010 to December 2011 at the Vietnam National Hospital of Pediatrics, Hanoi, Vietnam. All children with pneumonia who met the following inclusion criteria for CMV-associated pneumonia were enrolled in the study: (1) severe pneumonia based on WHO criteria (in infants <2 months old, severe chest in-drawing or a respiratory rate >60 per minute; in children ≥2 months–5 years, chest in-drawing [9]), (2) CMV-DNA detected in tracheal aspirates and blood plasma specimens with more than 5 × 10^3^ copies/mL[10], (3) not have specific immunocompromised status such as HIV infection, post-transplantation, known immunosuppressive disorder or prior chemotherapy, (4) no co-infection with bacteria or viruses other than CMV, and (5) consent from parents or legal guardian to participate in the study.

### Assessment of immune status

The immune status of the patients was assessed by the percentage of CD4-positive T lymphocytes for cellular immunity and serum IgG concentration for humoral immunity. In this study, when CD4 cells were <35% of total lymphocytes the CD4 percentage was considered low [11] and less than 176 mg/dL of IgG was defined as low serum IgG [12].

### Detection of viruses and bacteria

Tracheal aspirates were taken on admission from all patients for bacterial culture, including a rapid test for respiratory syncytial virus (RSV), influenza virus type A (FluA) and type B (FluB) (Standard Diagnostics, Korea) and RT-PCR for adenovirus (AdV) (Qiagen, Germany) and human rhinovirus (HRV) (Qiagen, Germany). CMV-DNA as detected from both tracheal aspirates and blood plasma using the real-time PCR method. The tracheal aspirates and EDTA-treated blood samples collected in the Respiratory Department were transferred to the Molecular Biology Laboratory, Microbiology Department, NHP. Tracheal aspirates and plasma specimens were stored at −70°C until tested.

Nucleic acid was extracted from 100 μL of each specimen by MagNAPure LC 2.0 robot (Roche Molecular Systems, Mannheim, Germany). The PCR amplification was performed in a total volume of 25 μL in the presence of TaqMan Universal PCR master mix (2X) (Qiagen, TaqMan MGB Probe, Germany), with each primer and TaqMan probe. PCR was performed with the IQ5 real-time PCR system (Bio-Rad, Hercules, CA) under the following conditions: 2 minutes at 46°C, 10 minutes at 95°C, 45 cycles of 95°C for 15 seconds and 58°C for 1 minute [13].

### Treatment

Children whose percutaneous oxygen saturation level was less than 92% received oxygen supplementation via facemask or nasal prongs. After taking the samples for bacterial culture, the patients were given 3rd or 4th generation cephalosporin antibiotics. After obtaining the disgnosis of CMV-associated pneumonia, we stopped the antibiotics and started anti-CMV treatment. All enrolled children were given ganciclovir (GCV) (Cymevene, manufactured by Roche, Germany) intravenously at a dose of 10 mg/kg/24 h for a duration of 14 days at most, followed by 5 mg/kg/24 h for 7 days or until CMV-DNA was not detected in blood plasma [14]. Intravenous immunoglobulin (IVIG) was given when the patients had low humoral immunity status. Clinical signs and symptoms were assessed daily. Complete blood count (CBC), hepatic and renal function, chest X-ray and CMV viral load were checked before the commencement of GCV treatment and then every 7 days until discharge. Treatment was considered successful if the patient no longer had respiratory symptoms and CMV-DNA in plasma was under the detection limit (<100 copies/mL).

### Statistical analysis

Data were entered into Epidata 3.1 (EpiData Association, Odense, Denmark), and then transferred into SPSS 10.0 (IBM SPSS, Armonk, NY, USA) for analysis. Continuous variables were tested for normal distribution with the Shapiro-Wilk statistic. Continuous variables were expressed as means +/− standard deviations for normally distributed variables or medians and interquartile ranges (IQR) for non-normally distributed variables. P < 0.05 was considered statistically significant.

### Ethical considerations

The Ethical Committee of the NHP approved the study. Written informed consent was obtained from the parents or legal guardians of the patients.

### Consent

**Figure 1:**
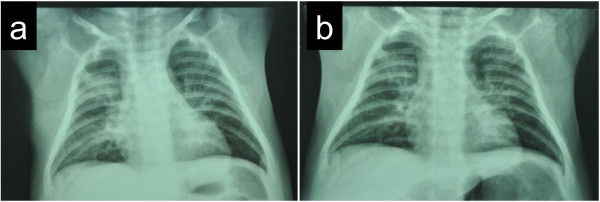
**Representative chest X-ray findings before and after treatment. (a)** X-ray on admission before treatment. **(b)** X-ray on day 14 of GCV treatment.

Written informed consent was obtained from the parents for publication of the X-ray (Figure 1).

## Results

### Patient profiles

During the study period, we enrolled 43 patients. The median age on admission was 57 (IQR 45–85) days. Twenty-six patients were boys (60.5%) and 17 (39.5%) were girls. In our study, 26 (60.5%) patients were the firstborn, 36 (83.7%) were born by vaginal delivery, 23 (53.5%) were exclusively breastfed and 25 (58.1%) were term infants.

The time interval between onset and admission was more than 7 days for 39 (90.7%) patients. Table 1 shows the main clinical and laboratory characteristics of the patients. Twenty-nine patients had anemia (Hb <11 g/dL) (67.4%) and 26 patients had hepatosplenomegaly on admission (60.5%). Thirty-nine patients had leukocytosis (>10,000/mm^3^) (90.7%), with mainly lymphocytes. Immune status was investigated in 36 patients. A low CD4 percentage (<35%) of T lymphocytes was observed in 25 of 36 (69.4%) patients and low serum IgG (<176 mg/dL) was observed in 9 of 35 (25.7%) patients. CMV-IgG was positive in all patients and CMV-IgM was positive in 29 (67.4%). The median CMV viral load in plasma was 59.5 (IQR 30–160) × 10^3^ copies/mL, whereas that in tracheal aspirate specimens was 120 (IQR, 7–400) × 10^3^ copies/mL on admission. The chest X-ray taken on admission showed interstitial infiltrate changes in 28 patients (65.1%). Twenty patients (46.5%) had combination interstitial and consolidation images. Only 5 (11.6%) patients had consolidation images.

**Table 1:** Clinical and laboratory information of the patients in this study

	**Number**	**Percentage**
Age, Median (IQR)	57 (45-85)days	
Male	26	60.5%
Female	17	39.5%
**History**		
Low birth weight (< 2,500 g)	16	37.2%
First-child	26	60.5%
Exclusive Breast fed	23	53.5%
Blood transfusion history	5	11.6%
Vaginal delivery	36	83.7%
Term infants (gestational age ≥38 weeks)	25	58.1%
**Symptoms**		
Weight (kg)	3.83 ± 0.95 (1.9-6)	
Malnourished	5	11.6%
Cough	43	100%
Diarrhea	10	23.3%
Wheeze	43	100%
**Signs**		
Body temperature ≥380C	23	58.5%
Sp02 <90% on room air	32	74.4%
Hepatomegaly	26	60.5%
**Laboratory test**		
CRP <0.6 mg/dl	36	83.7%
Hemoglobin <11 g/dl	29	67.4%
Leukocytosis >10,000 leukocyte /mm^3^	39	90.7%
Aspartate aminotransferase (AST) > 40 IU/L	32	74.4%
Alanine aminotransferase (ALT) >37 IU/L	19	41.2%
Immunological data		
Positive for CMV-IgM	29	67.4%
Positive for CMV-IgG	43	100%
Low CD4 (<35% of T cell )*	25/36	69.4%
Low IgG level (<176 mg/dl)**	9/35	25.7%
CMV viral load		
CMV-DNA copies/mL in plasma, Median (IQR), 10^3^ copies/mL	59.5 (30-160)	
CMV-DNA copies/mL in tracheal aspirates, Median (IQR), 10^3^ copies/mL	120 (7-4,000)	
Chest X-ray		
Interstitial changes	28	65.1%
Interstitial and consolidation	20	46.5%
Consolidation	5	11.6%
Time interval between onset and visit ≥7 days, Median (IQR)	15(10-21)	
**Outcome**		
Discharged	42	97.7%
Died	1	2.3%

*IQR* Interquartile range, *CMV* cytomegalovirus, *Data were available for 36 patients.**Data were available for 35 patients.

### Results for risk factors

We conducted subanalyses of risk factors for term versus pre-term infants, low-birth-weight versus normal-birth-weight infants, vaginal delivery versus cesarean delivery infants, and breastfed versus not breastfed infants. There were no differences in the viral load improvement between these subgroups (P > 0.05).

### Characteristics for each age group

The median length of hospitalization was 23 (IQR 18–37) days. The median duration of GCV treatment and oxygen treatment were 12 (IQR 7–21) days and 10 (IQR 3–19) days, respectively. The patients were divided into three groups according to their age: 30–60 days, 61–90 days and >90 days. There were imbalances in the number of patients in the age groups. The characteristics in each group are shown in Table 2. Twenty-five patients (58.1%) were 30–60 days old. The median value of CMV viral load in plasma was highest in the youngest group (30–60 days). The duration of GCV treatment decreased with increasing age (P < 0.05). The duration of both GCV treatment and oxygen treatment were higher in the oldest group (>90 days). As for immune status, a low CD4 percentage was observed in 81% of the youngest group (30–60 days).

**Table 2:** Characteristics of the patients in each age-group

**Age group**	**30-60 days**	**61-90 days**	**>90 days**	**P**
Number of patients (male/female)	25(15/10)	11(7/4)	7(4/3)	
CMV copies/mL in plasma, Median (IQR)	140(30-200)x 10^3^ copies/mL	90(42-200) x 10^3^ copies/mL	50(25-60) x 10^3^ copies/mL	0.22
CMV copies/mL in tracheal aspirates, Median (IQR)	66.9(5-396)x 10^3^ copies/mL	185(122-900) x 10^3^ copies/mL	212(15-410) x 10^3^ copies/mL)	0.53
SpO2 on admission, Median (IQR)	88(85-89)%	89(84-92)%	90(86-92)%	0.08
Oxygen treatment duration , Median (IQR)	12(6-22)days	7(0-13)days	9(0-22)days	0.29
GCV treatment duration , Median (IQR) *	13 (9-17)days	14(10-17)days	8(7-10) days	0.013*
Percentage of the patients with low IgG (IgG < 176 mg/dl)*	20%	33%	33%	0.02*
Percentage of the patients with low CD4 (CD4 < 35%)*	81%	56%	50%	0.001*

*p < 0.05 *IQR* Interquartile range.

### Clinical symptoms and laboratory data

After GCV treatment had started, fever decreased to a normal range within 2.5 (IQR 2–5) days and cough reduced within 5 (IQR 2–7) days. On admission, 29 patients had Hb levels under 11 g/dL. Only one patient needed a blood transfusion because the Hb level decreased from 9.2 g/dL to less than 8 g/dL. The anemia in these patients improved after GCV treatment. Thirty-nine patients had an elevated count on admission, which decreased gradually to within the normal range. No patient had a WBC decrease below the normal range (Table 3). Concentrations of liver enzymes (AST and ALT) were slightly elevated in 32 patients on admission. Though the concentrations of both enzymes increased transiently in 10 patients after 1 week of treatment, they returned to the same levels as before treatment at the end of treatment (Table 3). During antiviral therapy, platelet counts, renal function and electrolytes were in the normal range. In the subanalysis there were no significant differences in WBC count or concentration of AST and ALT changes between the subgroups (P > 0.05).

**Table 3:** Number of white blood cell (WBC), the concentrations of AST and ALT after ganciclovir (GCV) treatment (IU/L)

	**On admission**	**After 1 weeks**	**After 2 weeks**	**After 3 weeks**
WBC (×10^3^ cells/mL)*	16.9 ± 7.1	14.5 ± 5.1	11.8 ± 3.6	10.8 ± 3.9
Aspartate aminotransferase (AST)*, IU/L	80.12 ± 50.06	85.78 ± 72.31	70.92 ± 41.98	73.14 ± 49.09
Alanine aminotransferase (ALT)*, IU/L	44.82 ± 31.8	57.52 ± 58.9	52.26 ± 26.5	47.16 ± 26.1

*The values were shown as means +/- standard deviations.

### CMV viral load

**Figure 2:**
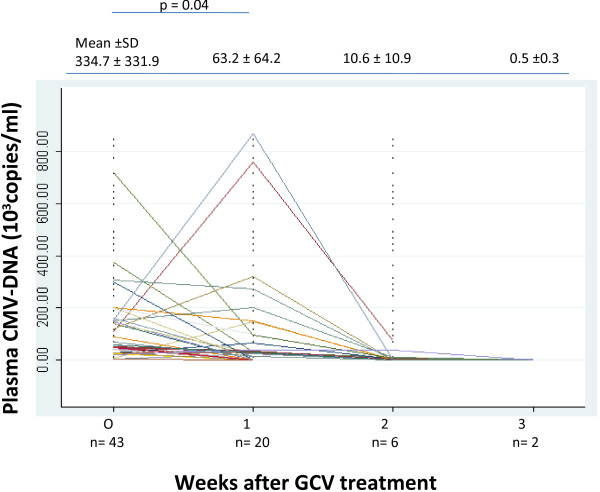
**Cytomegalovirus (CMV) viral load and the course of ganciclovir (GCV) treatment.** After 1-week treatment with GCV, CMV viral load reduced significantly (p < 0.05). The values are shown as means +/- standard deviations.

The median level of CMV viral load on admission was 334.7 ± 331.9 × 10^3^ copies/mL. Plasma CMV viral load declined rapidly after starting GCV treatment. After the first week, CMV-DNA fell under the detection limit (UDL) in 23 (53.5%) patients. These data were statistically significant with P < 0.05. CMV-DNA were UDL in 14 (32.6%) additional children at the end of the second week and in the 4 (9.3%) remaining children at the end of the third week. The decline of plasma CMV viral load is shown in Figure 2.

### Chest X-ray

The chest X-ray images improved with GCV treatment (Figure 1). After the GCV treatment, 37 patients (86.0%) showed a normal chest X-ray. As for the other 6 patients, a few lesions persisted on the chest X-ray on discharge, though they improved clinically as CMV-DNA became undetectable. Follow-up chest X-rays 1 month after discharge were normal.

### Outcome

Forty-two of 43 patients (97.7%) were cured and healthy when discharged. One patient acquired *Burkholderia cepacia* and developed respiratory failure and multiorgan failure, and died.

## Discussion

In this report, we present the clinical and laboratory features of and the effect of antiviral treatment on CMV-associated pneumonia in apart children without specific immunocompromised status such as HIV infection, post-transplantation, known immunosuppressive disorder or prior chemotherapy. Most of our patients were born by vaginal delivery, exclusively or partly breastfed and were term infants, but there were no statistically significant differences in changes in viral load between these subgroups of patients (P > 0.05). In Figure 2, there were four patients who had an increase in plasma viral load after one week of GCV treatment, but they were negative at the end of the second week. They were all female, of normal birth weight, full term, and exclusively breastfed. Two patients were delivered vaginally. We presume patients acquired infection after birth and infection progressed naturally. A limitation of our study is that it is difficult to know when the patients were exposed to CMV (for example, by intrauterine infection, vaginal infection, or breastfeeding). All developed symptoms of pneumonia after 30 days of birth so we concluded that they acquired CMV infection after birth because postnatal CMV pneumonia develops 4–8 weeks after birth [14]. Numazaki et al. [15] and Jim et al. [16] showed that a high human CMV viral load and prolonged virus excretion in breast milk were maternal risk factors for viral transmission to very low-birth-weight infants.

It was remarkable that 67.4% of our patients had anemia with a Hb level less than 11 g/dL and 60.5% had hepatosplenomegaly on admission (Table 1). These characteristics are not commonly seen in cases of pneumonia because of other causes. Our findings were consistent with Avila-Agüero et al., who showed 100% of patients had anemia and 82% had hepatomegaly [7] and who were also reported to have active CMV infection based on standard criteria [14]. In addition to this, 90.7% of patients had leukocytosis, mainly lymphocytosis, which was consistent with the study by Capulong et al. [2]. Generally, in cases of viral pneumonia, the number of WBCs in blood is normal or decreased.

The main finding from chest X-rays was severe bilateral interstitial change, which is one of the characteristics of interstitial pneumonia including CMV pneumonia [17]. However, it was impossible to diagnose CMV pneumonia solely based on by the chest X-ray findings. Because of limitations in our laboratory facilities, it was also difficult to exclude other respiratory viruses, such as influenza, parainfluenza, adenovirus, enterovirus, and human metapneumovirus, which also cause interstitial pneumonia.

It has been the consensus among researchers around the world that CMV infection is an opportunistic infection and CMV disease occurs primarily in immunocompromised patients. Generally, CMV infection is an inapparent infection for an immunocompetent patient. However, all patients in this study were apparently immunocompetent. The immune statuses of 36 patients (83.7%) were investigated. The levels of IgG decreased in 9 of 35 (25.7%) and the percentage of CD4-positive T cells decreased in 25 of 36 (69.4%) patients. As shown in Table 2, there was an imbalance in the number of patients in age groups and 81% of the patients 30–60 days old showed a low immune status with a low CD4 percentage according to the pediatric test reference value of the Mayo Clinic (P = 0.001) [11]. Those patients also had a longer GCV treatment duration (P = 0.013), and low IgG level (P = 0.02). One hypothesis to explain the smaller number of patients older than 90 days would be that as children grow, the immune system matures enough to protect them from CMV infection. The decreases observed were not so severe as to suggest congenital immunodeficiency. If they had severe congenital immunodeficiency, there would be more complications, with other opportunistic infections. Though other reflections of immune status such as natural killer cell activity should be examined, we suspected that other immune system problems were temporary, but related to the development CMV infection and pneumonia.

In our study, the CMV viral load in tracheal aspirate specimens on admission was higher than that in plasma specimens, which has been also reported in other studies [10,18]. The CMV-DNA copy number in plasma indicated the amount of free CMV-DNA circulating in the bloodstream. Active CMV replication is considered useful in monitoring the clinical course of CMV infections [10]. However, CMV-DNA copy number in tracheal aspirates indicated the total amount of free CMV-DNA and CMV-DNA in cells detached from the respiratory airway. In this study, high copy numbers of CMV-DNA were detected both in plasma and in the tracheal aspirates of each patient, which suggested that CMV replicated actively in the lungs of the patients and caused pneumonia.

GCV reduced CMV-DNA promptly in the plasma to below the detection limit, improving respiratory symptoms and chest X-ray images, which indicated the efficacy of GCV in this study. The effectiveness of GCV in the treatment of CMV infection has been reported in other series [2,3,7,19]. In our study, 97.7% had a good response to GCV therapy. There was one death (2.3%), in a patient with dual infection with *Burkholderia cepacia*. Capulong et al. reported that the mortality rate directly due to CMV pneumonia was 50% after renal transplantation [2], which was much higher than the rate in our study.

Our results also showed that GCV treatment is safe for severe CMV-associated pneumonia in children. Though GCV liver toxicity has been reported to be very severe [20], it was mild and temporal in our study. As shown in Table 3, liver enzymes increased slightly and transiently after GCV treatment. Renal function remained normal during treatment. Our results are different from reports using GCV in immunocompromised adult patients, where toxicity is frequently encountered, with manifestations of myelosuppression, hematologic abnormalities (primarily neutropenia, anemia, and thrombocytopenia), infusion site reactions, gastrointestinal abnormalities and central nervous system abnormalities [19,20]. Only one patient suffered from severe anemia with a Hb level less than 8 g/dL after the first week of antiviral treatment, which required red blood cell transfusion. We did not find any major toxicity associated with GCV treatment.

## Conclusions

From our results, we conclude that GCV is effective and safe in the treatment of severe CMV-associated pneumonia in children. However, a randomized clinical trial would be necessary to obtain a more conclusive recommendation for GCV treatment in CMV-associated pneumonia in children.

## Abbreviations

GCV: Gancyclovir; CMV: Cytomegalovirus; NHP: National Hospital of Pediatrics; IQR: Interquartile ranges; Hb: Hemoglobin level; WBC: White blood cell count; UDL: Under detection limit.

## Competing interest

The authors declare that they have no competing interests.

## Authors’ contributions

TD designed the study, treated the patients, collected data, did the statistical analysis and drafted the manuscript. LN conceived of and participated in the design of the study and reviewed and edited the draft manuscript. TP carried out the molecular genetic studies, SP performed the statistical analysis and HP helped to draft the manuscript. All authors read and approved the final manuscript.

## Pre-publication history

The pre-publication history for this paper can be accessed here:

http://www.biomedcentral.com/1471-2334/13/424/prepub
